# Whole genome and in-silico analyses of G1P[8] rotavirus strains from pre- and post-vaccination periods in Rwanda

**DOI:** 10.1038/s41598-020-69973-1

**Published:** 2020-08-10

**Authors:** Sebotsana Rasebotsa, Peter N. Mwangi, Milton T. Mogotsi, Saheed Sabiu, Nonkululeko B. Magagula, Kebareng Rakau, Jeannine Uwimana, Leon Mutesa, Narcisse Muganga, Didier Murenzi, Lisine Tuyisenge, Jose Jaimes, Mathew D. Esona, Michael D. Bowen, M. Jeffrey Mphahlele, Mapaseka L. Seheri, Jason M. Mwenda, Martin M. Nyaga

**Affiliations:** 1 Next Generation Sequencing Unit, Division of Virology, Faculty of Health Sciences, University of the Free State, Bloemfontein, 9300 South Africa; 2 Diarrhoeal Pathogens Research Unit, Sefako Makgatho Health Sciences University, Medunsa, Pretoria, 0204 South Africa; 3 Kigali University Teaching Hospital, College of Medicine and Health Sciences, University of Rwanda, P.O. Box 4285, Kigali, Rwanda; 4 Division of Viral Diseases, NCIRD, Centers for Disease Control and Prevention, Atlanta, GA 30333 USA; 5 World Health Organization, Regional Office for Africa, P.O. Box 06, Brazzaville, Republic of Congo

**Keywords:** Computational biology and bioinformatics, Evolution, Microbiology, Molecular biology

## Abstract

Rwanda was the first low-income African country to introduce RotaTeq vaccine into its Expanded Programme on Immunization in May 2012. To gain insights into the overall genetic make-up and evolution of Rwandan G1P[8] strains pre- and post-vaccine introduction, rotavirus positive fecal samples collected between 2011 and 2016 from children under the age of 5 years as part of ongoing surveillance were genotyped with conventional RT-PCR based methods and whole genome sequenced using the Illumina MiSeq platform. From a pool of samples sequenced (n = 158), 36 were identified as G1P[8] strains (10 pre-vaccine and 26 post-vaccine), of which 35 exhibited a typical Wa-like genome constellation. However, one post vaccine strain, RVA/Human-wt/RWA/UFS-NGS:MRC-DPRU442/2012/G1P[8], exhibited a RotaTeq vaccine strain constellation of G1-P[8]-I2-R2-C2-M2-A3-N2-T6-E2-H3, with most of the gene segments having a close relationship with a vaccine derived reassortant strain, previously reported in USA in 2010 and Australia in 2012. The study strains segregated into two lineages, each containing a paraphyletic pre- and post-vaccine introduction sub-lineages. In addition, the study strains demonstrated close relationship amongst each other when compared with globally selected group A rotavirus (RVA) G1P[8] reference strains. For VP7 neutralization epitopes, amino acid substitutions observed at positions T91A/V, S195D and M217T in relation to the RotaTeq vaccine were radical in nature and resulted in a change in polarity from a polar to non-polar molecule, while for the VP4, amino acid differences at position D195G was radical in nature and resulted in a change in polarity from a polar to non-polar molecule. The polarity change at position T91A/V of the neutralizing antigens might play a role in generating vaccine-escape mutants, while substitutions at positions S195D and M217T may be due to natural fluctuation of the RVA. Surveillance of RVA at whole genome level will enhance further assessment of vaccine impact on circulating strains, the frequency of reassortment events under natural conditions and epidemiological fitness generated by such events.

## Introduction

Group A rotavirus is a significant viral etiological agents of acute gastroenteritis (AGE) resulting in approximately 125,000 deaths annually in children under 5 years worldwide^1^. Rwanda rolled out the RotaTeq (Merck and Co., Whitehouse Station, NJ, USA) vaccine into her Expanded Program on Immunization in May 2012 and the vaccination coverage rate has been consistently high over the years > 95% since 2013, resulting in significant reduction in RVA-associated hospitalization among children younger than 5 years old^2^. Specifically, in 2013 and 2014 post introduction of the vaccine, 49% and 48% decreases in gastroenteritis cases requiring hospitalization, respectively, and 61% and 70% decreases in RV-specific diagnosis were observed, respectively^3^.

The RVA are double-stranded RNA viruses with a segmented genome enclosed within a triple-protein layer. The 11-gene segments code for six structural proteins (VP1–VP4, VP6 and VP7) and depending on the strain, five or six non-structural proteins (NSP1–NSP5/6)^4^. A more complex RVA classification is in use based on the genotype properties of all the 11 gene segments as Gx-Px-Ix-Rx-Cx-Mx-Ax-Nx-Tx-Ex-Hx which encodes for VP7-VP4-VP6-VP1-VP2-VP3-NSP1-NSP2-NSP3-NSP4-NSP5/6 proteins (x represents the genotype number)^5^. The segmented nature of RVA enables reassortment events that drive RVA evolution along with genomic rearrangement, genetic drift, deletion of gene sequences and zoonotic transmission^6–8^. Globally, majority of the human RVA strains possess either the Wa-like genotype constellation (I1-R1-C1-M1-A1-N1-T1-E1-H1) or the DS-1-like genotype constellation (I2-R2-C2-M2-A2-N2-T2-E2-H2)^9–11^. Currently, 36 G, 51 P, 26 I, 22 R, 20 C, 20 M, 31 A, 22 N, 22 T, 27 E and 22 H genotypes have been approved by the Rotavirus Classification Working Group (https://rega.kuleuven.be/cev/viralmetagenomics/virus-classification). Of the six common RVA genotypes (G1P[8], G2P[4], G3P[8], G4P[8], G9P[8] and G12P[8]) circulating globally, the G1P[8] genotype constitutes most of the human RVA infections^12^. In Africa, the G1P[8] genotype accounts for approximately 29% of all the circulating RVA strains^12^.

To date, four oral live attenuated RVA vaccines, Rotarix (GlaxoSmithKline, Rixensart, Belgium), RotaTeq (Merck and Co., Whitehouse Station, NJ, USA), ROTAVAC (Bharat Biotech, India) and ROTASIL (Serum Institute of India Pvt. Ltd., India) have been prequalified for global use by World Health Organization (WHO)^13^. All these vaccines are reported to be safe and highly efficacious based on clinical trial evaluations. In 2009, WHO recommended routine immunization of RVA vaccine to all infants to reduce the high mortality associated with RVA infections^14^. Rwanda was the first low-income country to introduce RotaTeq vaccine in May 2012 but switched to Rotarix in April 2017^15^. RotaTeq is an oral live attenuated RV vaccine comprising 5 bovine-human RVA reassortant strains (G1P[5], G2P[5], G3P[5], G4P[5] and G6P[8]) with a WC3 bovine backbone. The genotypes G1, G2, G3, G4 and P[8] are human rotavirus derived, while genotypes G6 and P[5] are bovine RVA derived^16^. RotaTeq is co-administered with other routine childhood vaccines in a three-dose schedule recommended at 2, 4 and 6 months of age^17^.

The rationale for RVA vaccine introduction was to combat the high morbidity and mortality of RVA-associated disease burden in Rwanda^18^. Rotavirus strain G1P[8] is the most prevalent genotype globally, several countries have analyzed the full genome of G1P[8] strains, however, Rwanda lacks such reports^8,19–24^. Amongst these studies, one investigated the whole gene sequences of G1P[8] RVA strains collected from the pre- and post-vaccination era in South Africa and the second investigated only G1P[8] strains collected during the post-vaccination era in Brazil^20,22^. Magagula and colleagues concluded that all the G1P[8] strains analyzed exhibited a Wa-like genetic constellation and shared a moderate nucleotide identity of 89–96% and 93–95% to Rotarix and RotaTeq G1P[8] vaccine components, respectively. In the second study, Santos and colleagues, reported double reassorments between Wa-and DS-1-like genogroups and also, Wa- and AU-1-like genogroups. A comparison of the amino acid residues present in the antigenic epitopes of VP7 and VP4, indicated a differences in the electrostatic charge distribution, between the Brazilian G1P[8] wild-types strains and Rotarix and RotaTeq G1P[8] vaccine strains. The availability of RVA strains collected from 2011 to 2016 in Rwanda during the pre- and post-vaccination periods with the RotaTeq vaccine presented an opportunity to assess vaccine impact on the molecular diversity of circulating strains. To gain insight into the overall genetic makeup and evolution of the G1P[8] strains from Rwanda, whole genome analyses was conducted and the data was compared with RVA strains from other parts of the world. To our knowledge, this study is the first to analyze circulating RVA strains in Rwanda on a whole genome level as compared to the traditional binary classification into G /P genotypes.

## Results

### Whole genome constellation analyses

**Figure 1:**
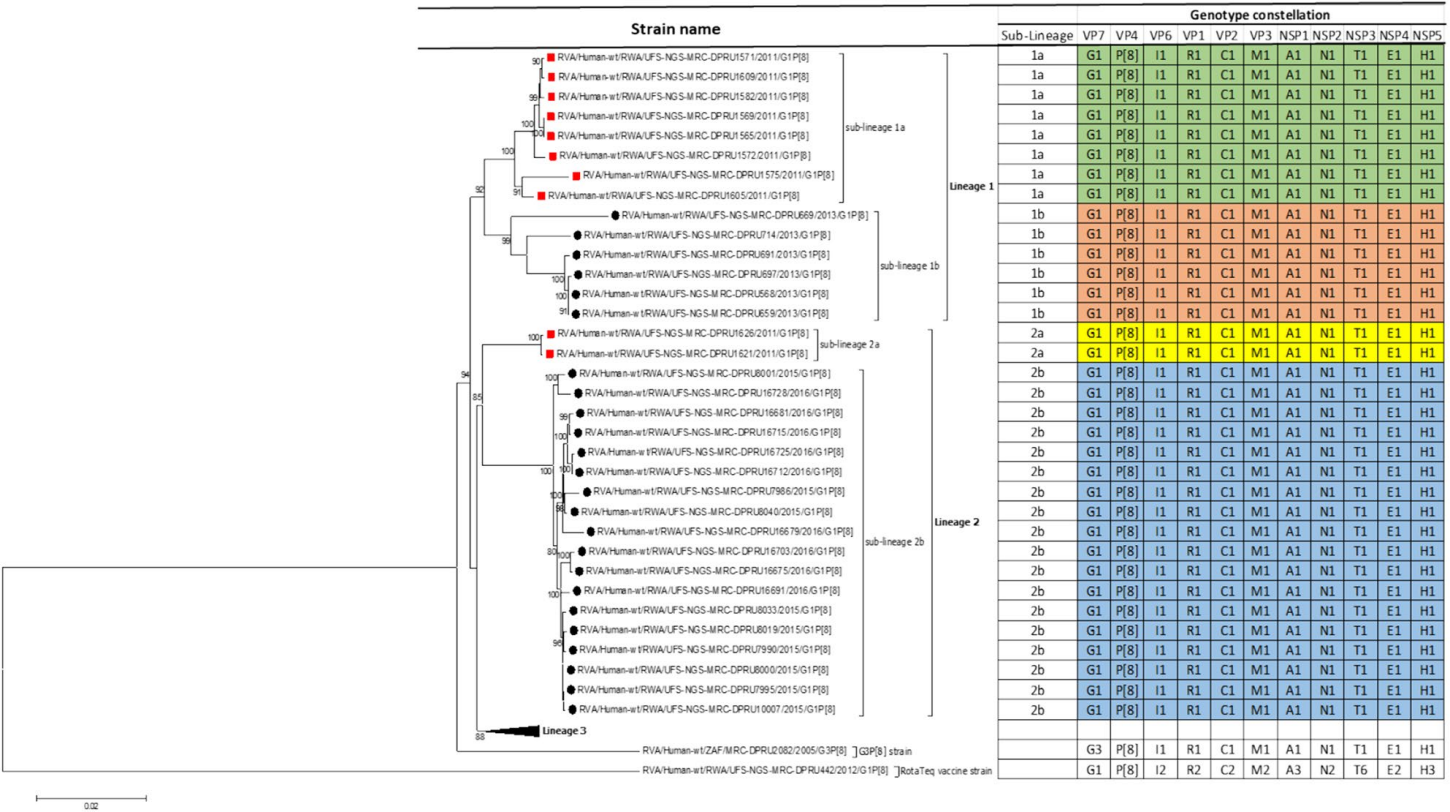
Maximum likelihood phylogram revealing the genetic relatedness of the concatenated, whole-gene ORF sequences (17,492 base pairs) for the 25 post-vaccine G1P[8] and 10 pre-vaccine G1P[8] RVA study strains characterized by whole-gene analysis. The strain-specific genotype constellations are included to the right of each strain and were identified using the assembled ORF sequences. Black circle denotes post-vaccine strains, while red squares denotes pre-vaccine strains. Bootstrap values ≥ 70% are indicated at branch nodes where applicable and the scale bar indicates the number of nucleotide substitutions per site. On the table, colored in yellow and sky blue are lineage 2 pre- and post-vaccine strains belonging in sub-lineage 2a and sub-lineage 2b, respectively. Colored in green and brown are lineage 1 pre- and post-vaccine strains belonging in sub-lineage 1a and sub-lineage 1b, respectively.

A pool of 158 randomly selected Rwandan stool samples were sequenced and only 36 were genotype G1P[8], which was the inclusion criteria for this study. The genome constellations of the 35 pre- and post-vaccine G1P[8] strains described in this study was the typical G1-P[8]-I1-R1-C1-M1-A1-N1-T1-E1-H1. One strain was a reassortant G1P[8] with genetic backbone of I2-R2-C2-M2-A3-N2-T6-E2-H3 (Fig. 1). The reassortant G1P[8] Rwandan strain was detected from a 2 months old infant collected 4 days after vaccination with the first dose of RotaTeq vaccine. Almost all gene segments of the reassortant G1P[8] strain shared a near absolute nucleotide and amino acid identity to cognate gene sequences of the RotaTeq vaccine G1P[8] strain. This suggested that the reassortant G1P[8] strain detected in a Rwandan child with diarrhoea was a RotaTeq vaccine derived G1P[8] (vdG1P[8]) strain. The lengths of the open reading frame (ORFs) for segments 1–11 for all 35 typical G1P[8] strains were 3,264 bp, 2,637 bp, 2,505 bp, 2,325 bp, 1,458 bp, 1,194 bp, 930 bp, 951 bp, 978 bp, 525 bp, and 594 bp, respectively. The ORF sequences for all 11 genes of these 35 Rwandan G1P[8] were sequenced and deposited in GenBank under accession numbers MN632673-MN633067 for all the gene segments.

### Phylogenetic and sequence analyses

**Figure 2:**
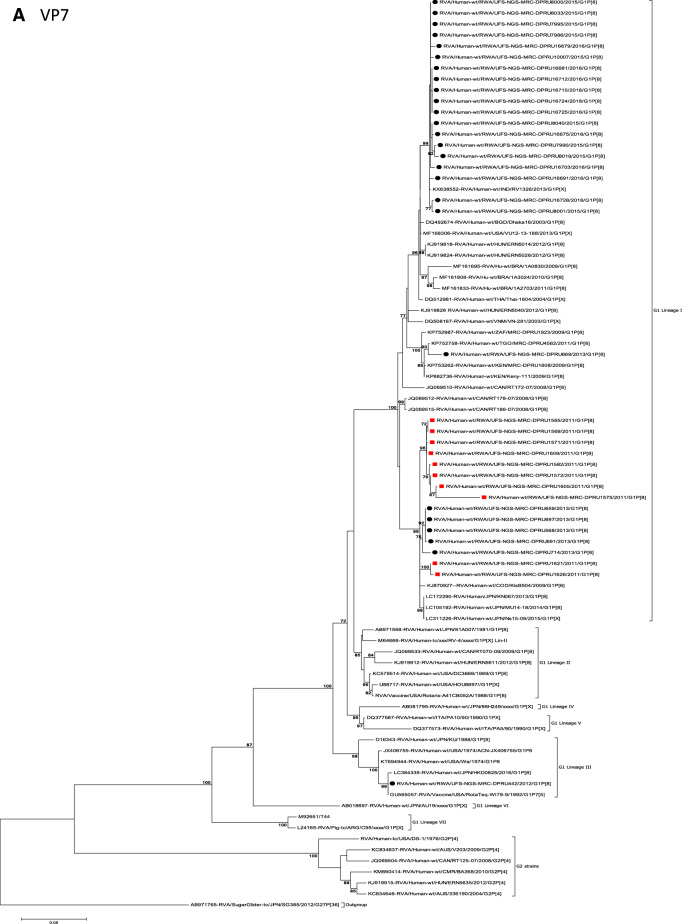

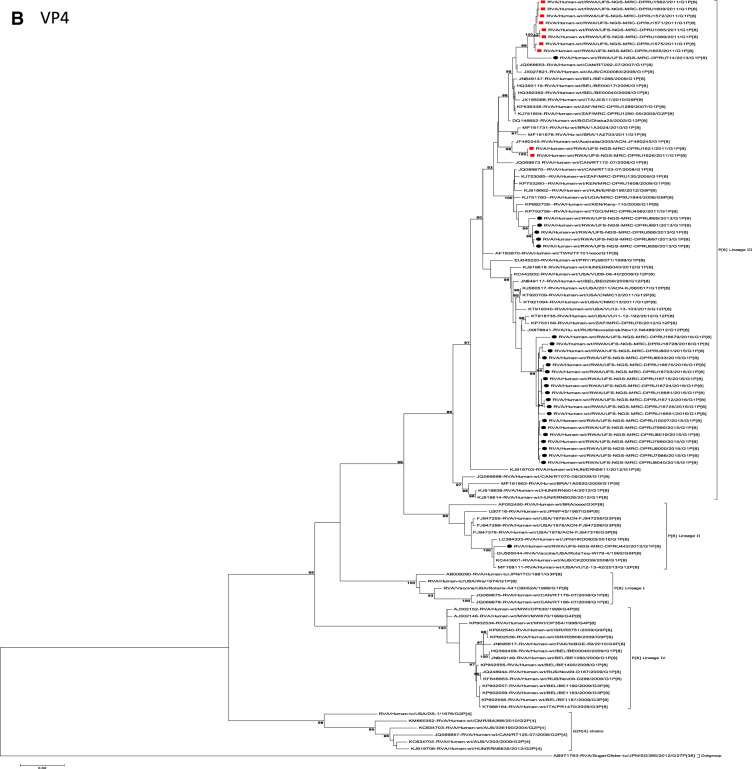

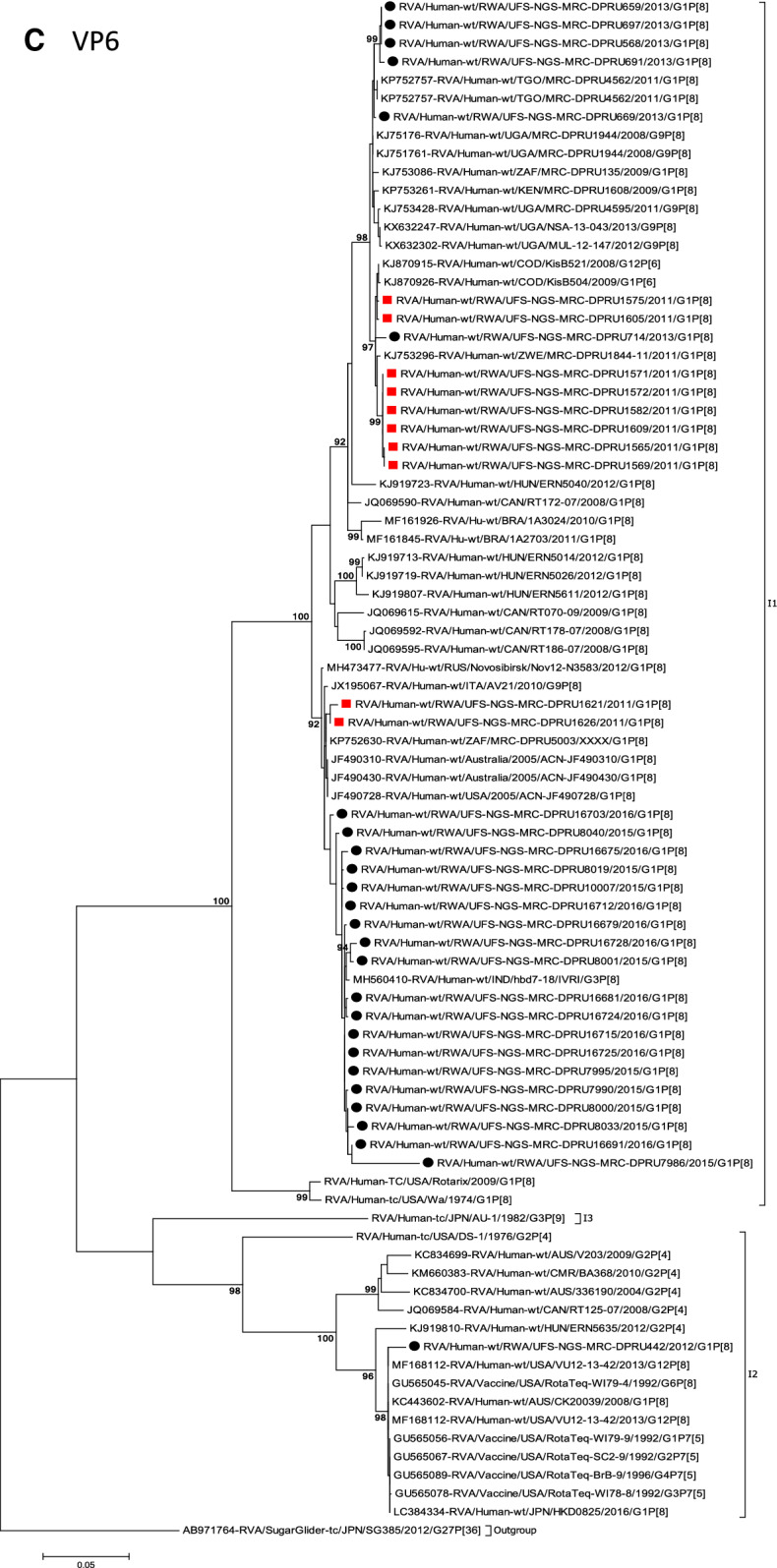

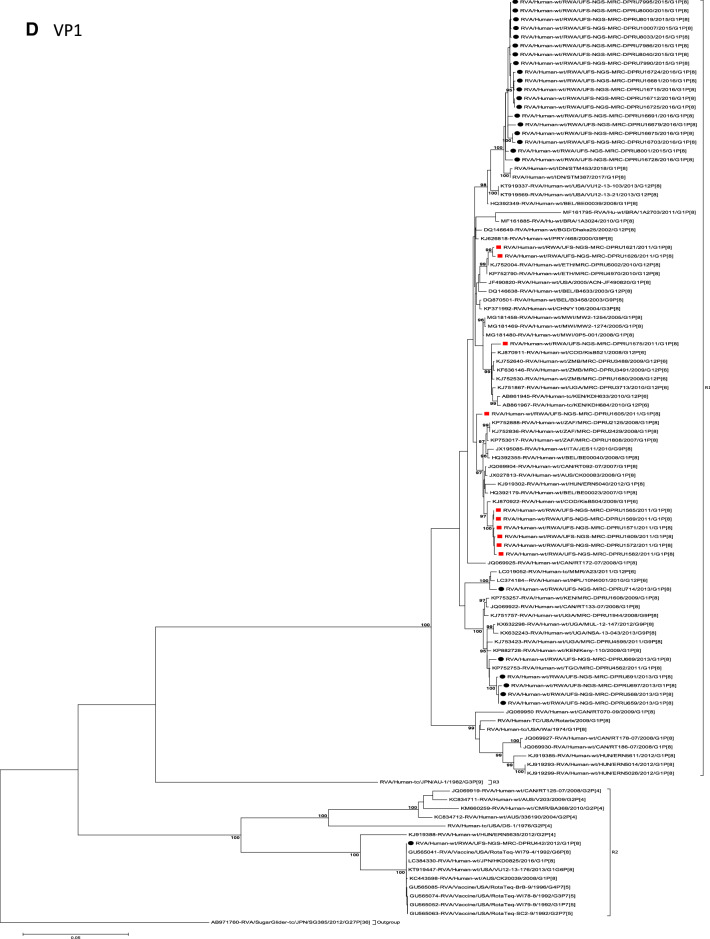

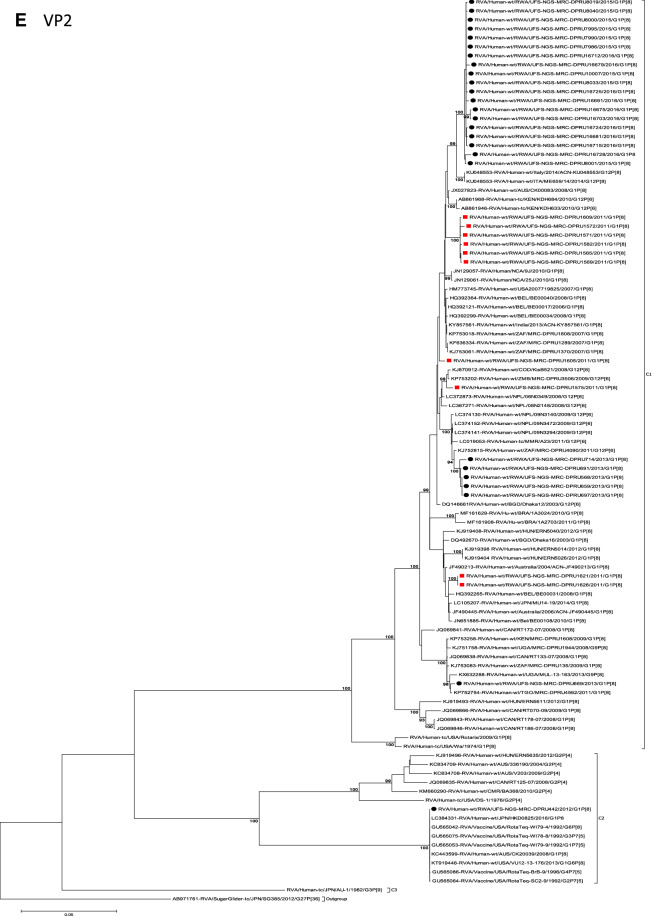

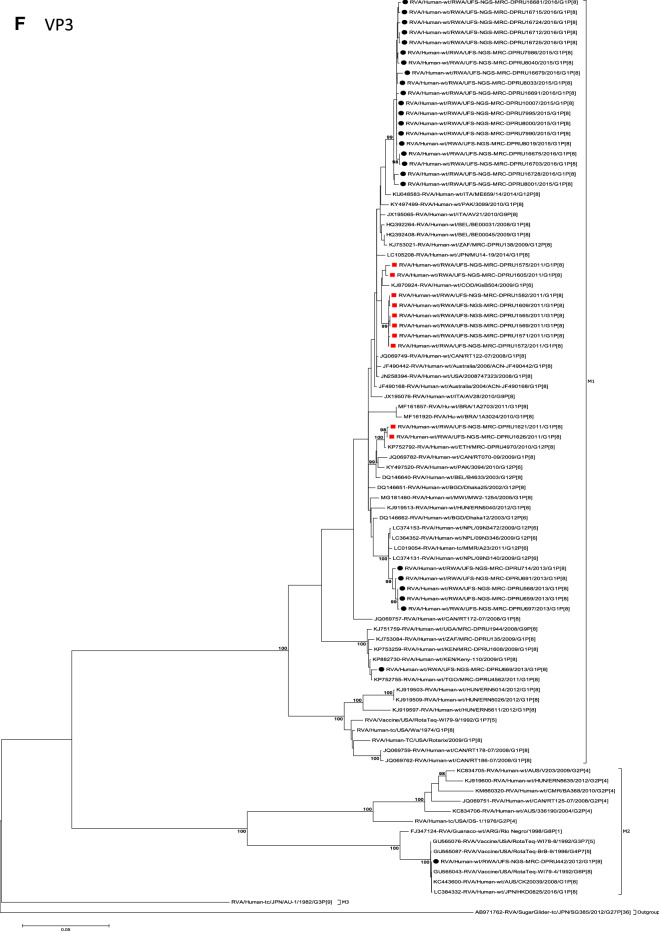

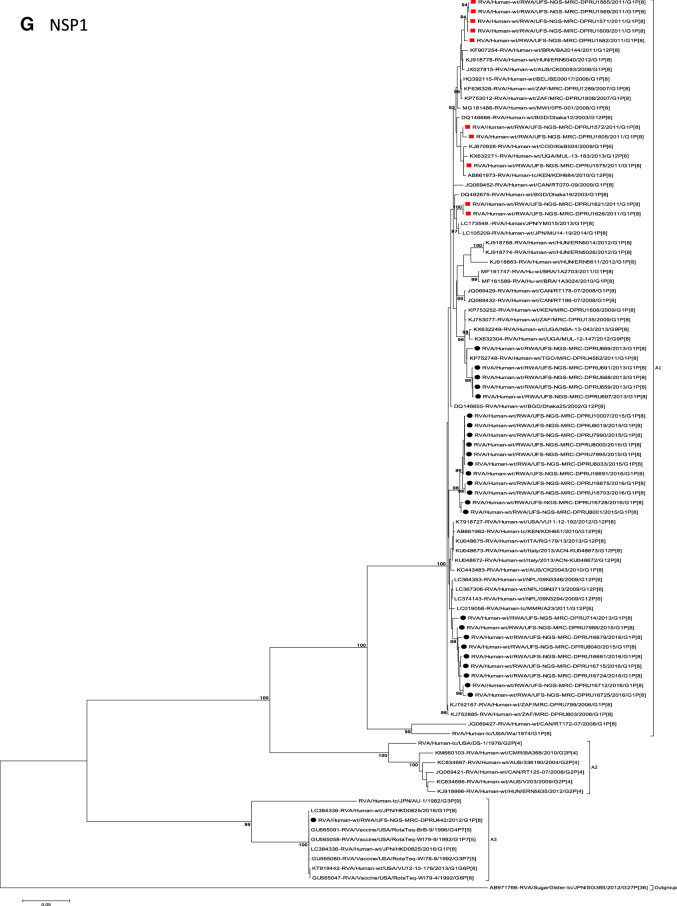

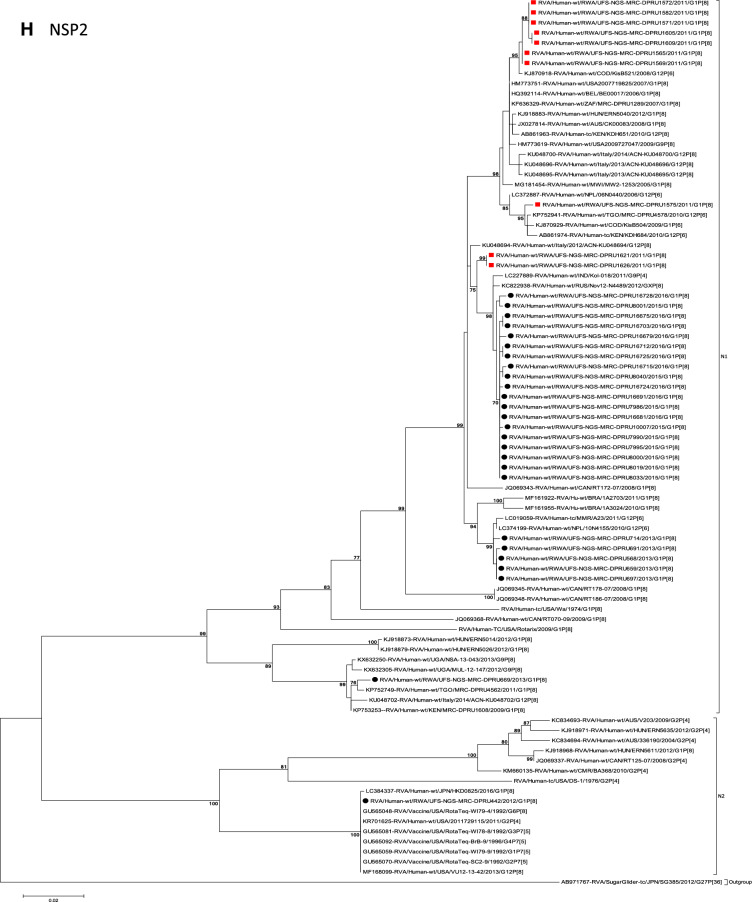

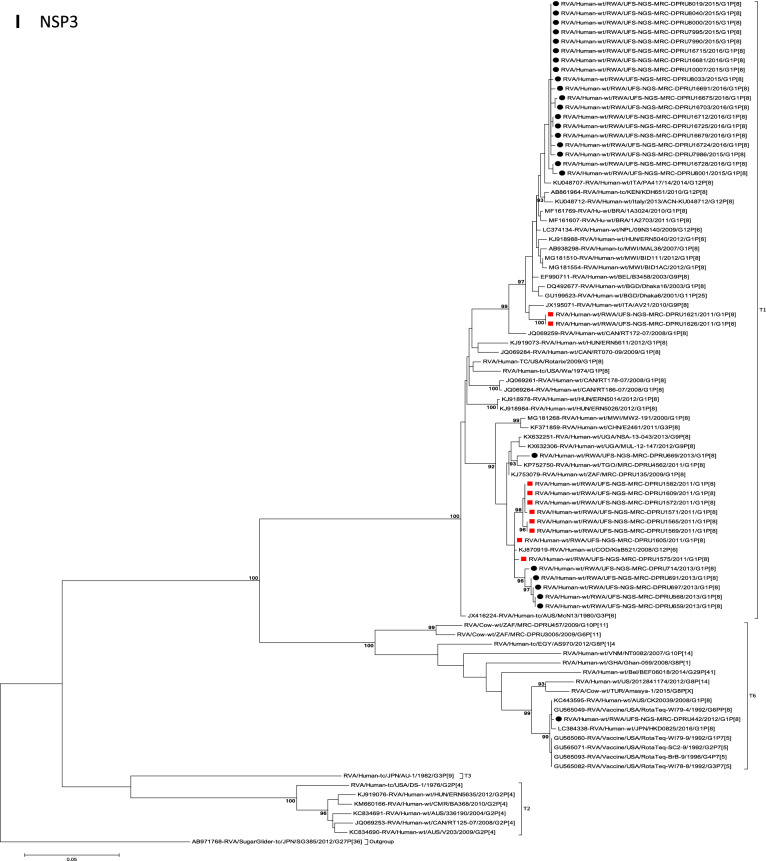

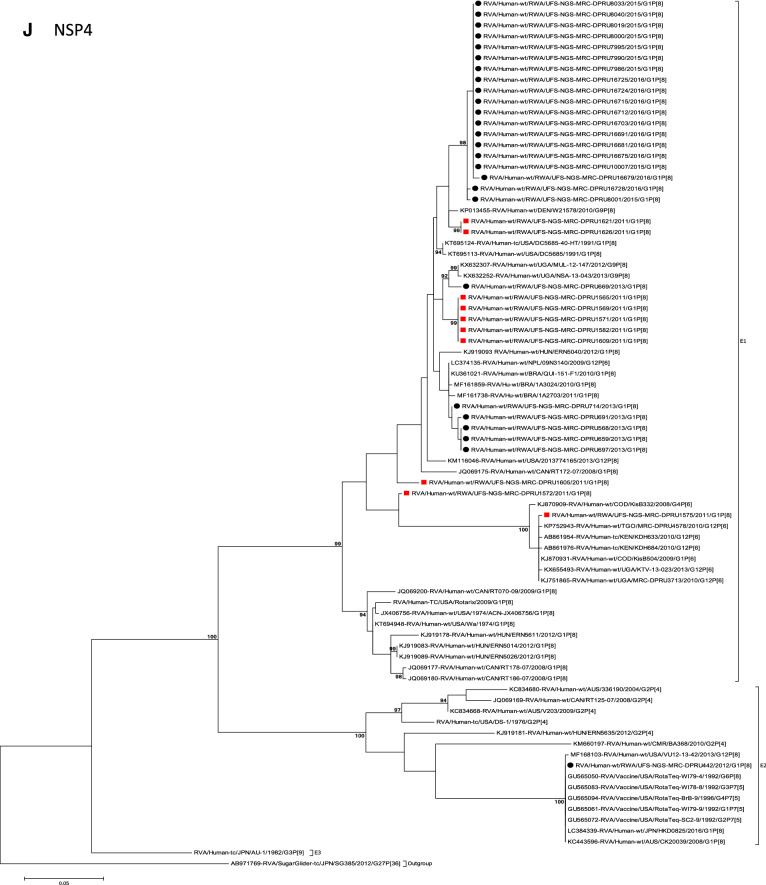

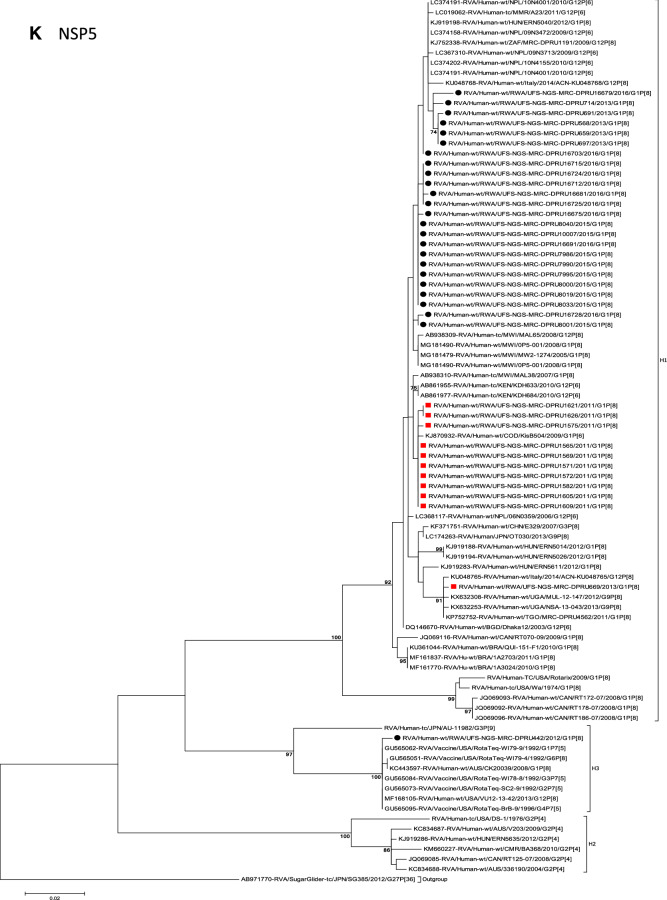
(**A**)–(**K**) Phylogenetic relatedness of rotavirus group A species base on (**A**) VP7, (**B**) VP4, (**C**) VP6, (**D**) VP1, (**E**) VP2, (**F**) VP3, (**G**) NSP1, (**H**) NSP2, (**I**) NSP3, (**J**) NSP4 and (**K**) NSP5 of the study strains from Rwanda with representatives of known human and animal rotavirus genotypes. Pre- and post-vaccine strains are indicated with a red squares and black circles, respectively. Bootstrap values ≥ 70% are indicated at each branch node. Scale bars represents substitutions per nucleotide site.

The concatenated ORF sequence analyses revealed the clustering of the Rwandan pre- and post-vaccine RVA strains into two lineages (Fig. 1). Within each lineage there were two paraphyletic sub-lineages with pre-vaccine introduction strains occupying one of the sub-lineages and post-vaccine introduction strains occupying the other (Fig. 1). Overall, the 35 typical pre- and post-vaccine introduction G1P[8] strains demonstrated a close genetic relationship amongst themselves as compared with other RVA G1P[8] strains circulating on a global scale (Fig. 2A–K). Even though Rotarix vaccine was not used in Rwanda at the time of the study, we included the Rotarix vaccine gene sequences in all the analyses.

### Phylogenetic and sequence analyses of VP7 gene

Phylogenetically, RVA G1 reference genotypes from human and animal RVA strains utilized in this analyses mapped into the seven known lineages (I–VII) and 35 of 36 Rwandan pre- and post-vaccine introduction G1P[8] strains clustered into lineage I, which consisted of a global collection of G1 strains detected during 2003–2015 RVA seasons^20,25^. The lineage I strains were further segregated into sub-lineages usually by year and in some cases by vaccination status. Within this lineage I, the G1 strains segregated into two sub-lineages: one consisted of 19 G1 strains detected in the post-vaccination era and the other was a mixture of both pre-vaccine (ten G1) and post-vaccine (five G1) introduction strains (Fig. 2A). The nucleotide (nt) and amino acid (aa) identities of the 19 G1 post-vaccine introduction strains amongst themselves was in the range of 99.2–100% and 98.0–100%, respectively, whereas the nt (aa) similarities of the five post- and ten pre-vaccine introduction mixed G1 sub-lineage was 94.7–100% (92.7–100%). Further comparison of the G1 strains in the typical post-vaccine sub-lineage with those in the mixed pre- and post-vaccine sub-lineage revealed a nt (aa) similarities of 92.7–96.3% (90.7–96.0%). The VP7 gene of Rwandan post-vaccination introduction strain RVA/Human-wt/RWA/UFS-NGS-MRC-DPRU669/2013/G1P[8] was an orphan gene that did not cluster in either the typical post-vaccine sub-lineage or mixed pre-and post-vaccine introduction sub-lineage (Fig. 2A). The VP7 gene sequence of the RotaTeq vdG1P[8] strain, RVA/Human-wt/RWA/UFS-NGS-MRC-DPRU442/2012/G1P[8], clustered into lineage III (Fig. 2A) alongside cognate gene sequence of the RotaTeq vaccine strain RVA/Vaccine/USA/RotaTeq-WI79-9/1992/G1P7[5] (Fig. 2A). The VP7 gene sequences of strain RVA/Human-wt/RWA/UFS-NGS-MRCDPRU442/2012/G1P[8] displayed absolute identity with the VP7 gene of RotaTeq reassortant strain, RVA/Vaccine/USA/RotaTeq-WI79-9/1992/G1P7[5] and appeared to be a RotaTeq VP7 gene.

### Comparative analyses of neutralizing antigenic epitopes in the VP7 proteins of Rwandan G1P[8] and vaccine strains of RVA

**Figure 3:**
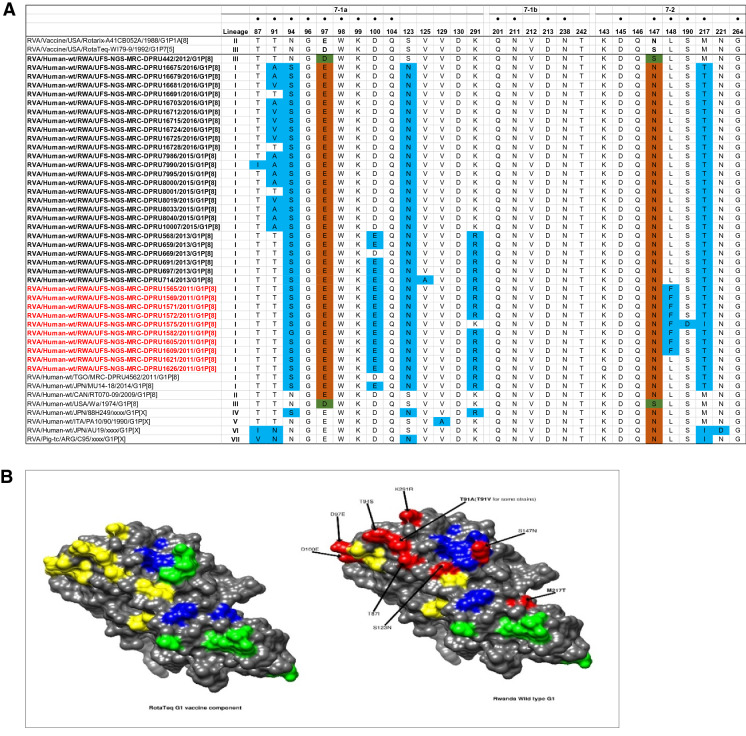
(**A**) The alignment of the G1 component of Rotarix and RotaTeq vaccines and Rwandan wildtypes circulating from 2011- 2016 RVA seasons, based on the three VP7 antigenic residues (7-1a, 7-1b, and 7-2). Amino acids residues at positions 97 and 147 differ between Rotarix and RotaTeq and are indicated in boldface. Study strains amino acid residues highlighted in sky blue differs from both Rotarix and RotaTeq, while the green and brown colored residues are different from Rotarix and RotaTeq, respectively. The black dot indicates changes in the residues associated with escape neutralization with monoclonal antibodies. Post-vaccine and pre-vaccine G1P[8] study strains are bolded in black and red, respectively. (**B**) Location of surface-exposed amino acids differences between VP7 protein of RotaTeq G1 vaccine component versus a G1 wild-type strain from Rwanda (indicated in red). Antigenic epitopes in 3B are colored in yellow (7-1a), green (7-1b), and blue (7-2).

Structurally, the VP7 gene contains two defined neutralization epitopes: 7-1 and 7-2^26^. Aoki and colleagues further subdivided 7-1 epitope into 7-1a and 7-1b^26^. These three antigenic epitopes comprise 29 amino acid residues (14 residues in 7-1a,6 residues in 7-1b and 9 residues in 7-2). Using the G1 alignment for the VP7 gene, we identified amino acid differences in these neutralization epitopes between the wild-type Rwandan G1 RVA strains and the RotaTeq and Rotarix vaccine G1 strain (Fig. 3A). Out of these 29 amino acid residues, 20 (amino acids G96, W98, K99, Q104, V129 and D130 in 7-1a region, all positions in 7-1b region and K143, D145, Q146, N221, G264 in the 7-2 region) were completely conserved among all the Rwandan G1 strains. Relative to the G1 component of the RotaTeq vaccine, the Rwandan G1 strains showed up to 10–11 differences (T87I, T91A/V, N94S, D97E, D100E, S123N, K291R, S147N, L148F and M217T) located on the surface of the protein structure RotaTeq (Fig. 3A,B). Among these changes seen relative to RotaTeq vaccine G1 strain, only the T91A/V, S190D and M217T substitution at positions 91, 190 and 217 involve a change from a polar to non-polar molecule, polar to negative molecule and non-polar to polar molecule, respectively, were radical in nature. The remainder of the changes seen were neutral or conservative in nature.

### Comparative analyses of cytotoxic T lymphocytes epitopes of the G1 proteins of Rwandan and vaccine strains of RVA

**Figure 4:**
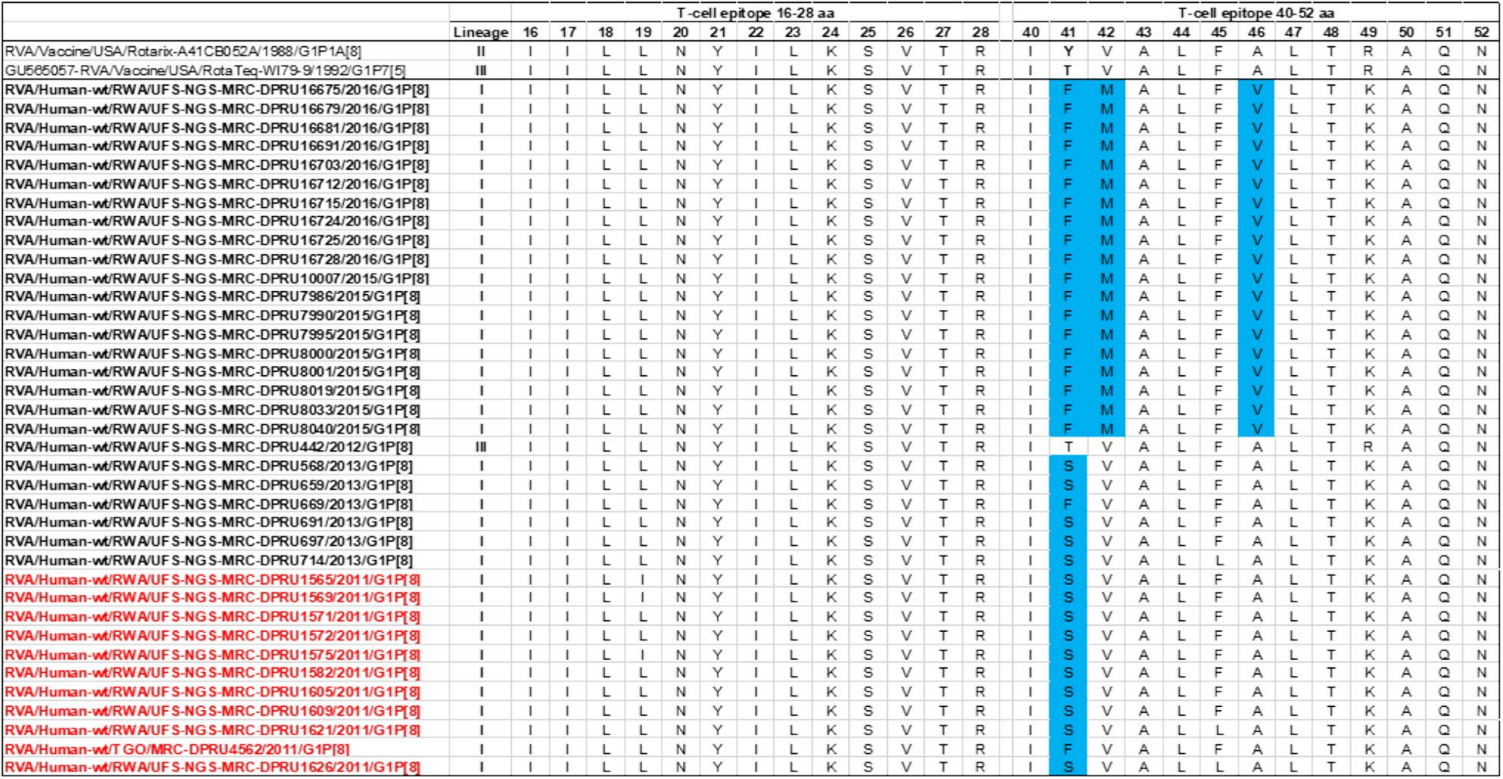
Alignment of antigenic residues in T-cell antigen epitopes of the G1 vaccine component contained in Rotarix and RotaTeq compared to Rwanda G1 wild type strains circulating from 2011–2016 RVA seasons. Amino acid changes are highlighted in sky blue.

Cytotoxic T lymphocytes (CTL) have been linked to clearance of RV infection resulting in complete short-term and partial long-term protection against re-infection^27^. Two linear CTL epitopes responsible for this activity have been reported on VP7 protein at amino acid positions 16–28 and 40–52^28,29^. An analyses of the Rwandan G1 pre- and post-vaccine introduction strains showed three amino acid differences at positions T41F/S, V42M and A46V with G1 component of the RotaTeq and Y41F/S, V42M and A46V to Rotarix vaccines (Fig. 4). Amongst these amino acid differences, the change at position T41F relative to the RotaTeq G1 gene sequence was radical in nature and resulted in a change in polarity from polar to nonpolar, while the other two substitutions were conservative in nature. Twenty-three out of the 26 amino acids that comprises the T-cell antigen epitopes were completely conserved.

### Phylogenetic and sequence analyses of VP4 genes

The VP4 gene sequences of the 35 Rwandan strains and a single RotaTeq vdG1P[8] strain collected in the pre- and post-vaccination periods were compared with representative human RVA from the four established VP4 P[8] genotype lineages (I to IV)^25,30^. With the exception of RotaTeq vdG1P[8] strain RVA/Human-wt/RWA/UFS-NGS-MRC-DPRU442/2012/G1P[8] which clustered in P[8]-lineage II with the P[8] component of RotaTeq vaccine strain RVA/vaccine/USA/RotaTeq-W-179-4/1992/G6P1A[8], the other 35 study strains clustered in P[8]-lineage III (Fig. 2B). Within P[8]-lineage III cluster, the Rwandan strains segregated into 3 typical and one mixed sub-lineages (Fig. 2B). The first sub-lineage consisted of 8 pre-vaccine introduction P[8] and a single post-vaccine introduction P[8] strains and they shared nt (aa) identities in the range of 99.7–100% (99.4–100%) amongst themselves (Fig. 2B). The second sub-lineage comprised of only 2 pre-vaccine introduction strains detected in 2011 RVA season and shared an absolute identity with each other (Fig. 2B). The third sub-lineage was characterised of 5 post-vaccine introduction strains circulating in the 2013 RVA season and shared a nt (aa) similarities amongst themselves ranging from 99.4–99.9% (98.6–100%) (Fig. 2B) and the fourth sub-lineage comprised of 19 post-vaccine introduction P[8] strains and shared nt (aa) identities amongst themselves in the range of 98.8–100% (98.0–100%) (Fig. 2B). Comparison of pre-vaccine introduction strains in the mixed sub-lineage to the post-vaccine introduction strains in the third and fourth sub-lineages revealed a moderate nt (aa) similarities in the range of 95.7–97.8% (89.5–94.5%). The VP4 P[8] component of the RotaTeq vdG1P[8] strain RVA/Human-wt/RWA/UFS-NGS-MRC-DPRU442/2012/G1P[8] clustered distinctly in P[8]-lineage II together with the RotaTeq vaccine strain RVA/Vaccine/USA/RotaTeq-WI79-4/1992/G6P1A8 and G3P[8] strains detected in 1976 in the US, G1P[8] strains detected in 2008 in Australia and G1P[8] detected in 2016 in Japan (Fig. 2B).

### Comparative analyses of neutralizing antigenic epitopes in the VP4 protein of Rwandan G1P[8] and vaccine strains of RVA

**Figure 5:**
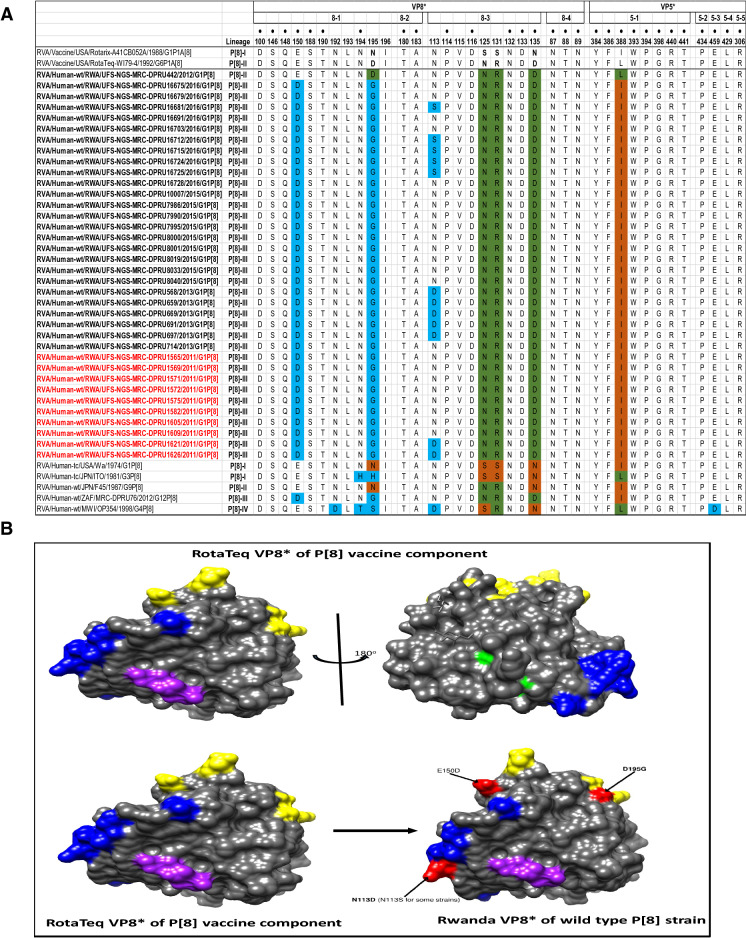
(**A**) The alignment of the P[8] component of Rotarix and RotaTeq vaccines and wild type P[8] strains circulating in Rwanda from 2011–2016 RVA seasons, based on the antigenic residues in VP4. VP4 is divided into two regions VP8* (antigenic epitope 8-1, 8-2, 8-3, and 8-4) and VP5* (antigenic epitope 5-1, 5-2, 5-3, 5-4, and 5-5). The black dot indicates changes in the residues associated with escape neutralization with monoclonal antibodies. Amino acids differences between Rotarix and RotaTeq are shown at positions 195 (antigenic epitope 8-1), 125, 131 and 135 (antigenic epitope 8-3) and are indicated in boldface. Study strains amino acid residues highlighted in green and brown are residues that are different from Rotarix and RotaTeq, respectively. Also, amino acids differences between study strains and Rotarix and RotaTeq are highlighted in sky blue. Post-vaccine and pre-vaccine G1P[8] study strains are bolded in black and red, respectively. **(B)** Location of surface-exposed amino acids differences between VP8* protein of RotaTeq P[8] vaccine component versus a wild-type P[8] strain from Rwanda (indicated in red). Antigenic epitopes in (**B**) are colored in yellow (8-1), green (8-2), blue (8-3) and purple (8-4).

The VP4 protein, which in this study represented P[8] strains, was divided into the VP8* and VP5* regions for comparison. The VP8* region contains four (8-1 to 8-4) neutralizing antigenic epitopes, while the VP5* region has five (5-1 to 5-5)^31^. These two epitopes contain 37 amino acid residues, 25 in the VP8* and 12 in the VP5* antigenic epitope regions. Out of these 37 amino acid residues that spans the neutralization epitopes, those at positions 100, 146, 148, 150, 188, 190 and 194 (8-1 region), 180 and 183 (8-2 region), 114, 116, 132, 133, and 135 (8-3 region), 87, 88, and 89 (8-4 region), 384, 386, 388, 393, 394, 398, 440, and 441 (5-1 region), 434 (5-2 region), 459 (5-3 region), 429 (5-4 region), and 306 (5-5 region), are known neutralization escape mutation sites (Fig. 5A)^32,33^. A comparison of the Rwandan P[8] strains to the P[8] component of RotaTeq and Rotarix vaccines showed 29 identical amino acid residues distributed through the antigenic epitopes of the VP4 gene (Fig. 5A). The differences between the P[8] study strains and vaccine P[8] component of RotaTeq and Rotarix were mostly contained in VP8* epitopes 8-1 and 8-3. Relative to the RotaTeq, the P[8] component of the Rwandan G1P[8] strains exhibited 3–4 amino acid differences. The changes identified relative to the RotaTeq P[8] vaccine strain were seen at positions E150D and D195G in neutralization epitope 8-1 region (Fig. 5A,B) and were all located on the surface of the protein structure. Overall, the amino acid changes identified at positions N113D, S131R and N135D, resulted in a change in charge from a polar molecule with potential side chain with five hydrogen bonds to a negatively charged molecule with four hydrogen bonds, and finally, amino acid substitutions at position N195G for both vaccines, resulted in a change in polarity.

### Phylogenetic and sequence analyses of VP1–VP3, NSP1, NSP4 and NSP5

These six genes segregated into typical pre- and post-vaccine introduction clusters, hence presented together. Phylogenetic analyses based on VP1–VP3, NSP1, NSP4 and NSP5 nucleotide sequences of each Rwandan G1P[8] strain showed that each gene (exception of RotaTeq vdG1P[8] strain) segregated and clustered with cognate gene sequences of Wa-like strains belonging to genotype 1 (Fig. 2D–G,J,K). The VP1 gene sequences of the 35 typical Rwandan G1P[8] strains segregated into a single pre-vaccine introduction sub-lineage exhibiting nt (aa) similarities in the range of 99.9–100% (99.8–100%) and 2 post-vaccine introduction sub-lineages with nt (aa) identities in the range of 96.8–98.0% (98.7–99.5%) (Fig. 2D). Four pre-vaccine introduction orphan strains that did not cluster in either of the three sub-lineages were also observed (Fig. 2D). For VP2, the C1 genes segregated into three typical sub-lineages (one typical pre- and two post-vaccine introduction) and five orphan strains which did not cluster into either of the three sub-lineages (Fig. 2E). The two post-vaccine introduction sub-lineage consisting of five and 19 strains shared nt (aa) similarities in range of 99.6–100% (99.6–100%) and 99.3–100% (99.7–100%), respectively. Furthermore, the typical pre-vaccine introduction sub-lineage incorporated strains (n = 6) detected in the 2011 RVA season and with nt (aa) sequence identities amongst themselves in the range of 99.9–100% (99.8–100%). Phylogenetically, the VP3 gene sequences of the Rwandan pre- and post-vaccine introduction strains separated into three typical sub-lineages and three orphan strains were observed (Fig. 2F). The first post-vaccine sub-lineage consisted of 19 strains and the second one had five strains that shared nt (aa) similarities ranging from 99.2–100% (98.9–100%) and 99.4–100% (99.3–100%), respectively. The pre-vaccine introduction sub-lineage contained strains that shared nt (aa) identities of 99.3–100% (98.5–100%). The NSP1 gene sequences of the typical Rwandan pre- and post-vaccine introduction G1P[8] strains were grouped into a single sub-lineage consisted of all ten pre-vaccine introduction strains and three typical post-vaccination introduction sub-lineages (Fig. 2G). The pre-vaccine strains shared nt (aa) similarities that ranged from 99.0–99.9% (97.5–99.8%). The three post-vaccine introduction NSP1 genes that segregated into three sub-lineages revealed nt (aa) identities of 98.7–99.9% (98.1–99.8%), 99.0–99.9% (98.4–99.8%) and 98.2–99.9% (98.1–99.8%), respectively. The NSP4 gene sequences of the Rwandan strains also segregated into two typical post-vaccination sub-lineages and a single pre-vaccination sub-lineage (Fig. 2J). Six orphan Rwandan E1 genotypes did not cluster into either the pre- or post-vaccine introduction sub-lineages (Fig. 2J). The first typical post-vaccine introduction strain sub-lineage comprised of 19 NSP4 genes and the second one had five E1 strains detected in 2011 RVA season which exhibited a nt (aa) identities in the range of 99.4–100% (99.4–100%) and 99.4–100% (99.4–100%), respectively. The pre-vaccination sub-lineage comprised of five NSP4 strains with absolute gene identities among themselves. Phylogenetically, the NSP5 gene did not resolve and the bootstrap support was not enough to separate the strains into distinct sublineages. On the other hand, a homologous relationship was observed for the VP1, VP2, VP3, NSP1, NSP4 and NSP5 gene segments of strain RVA/Human-wt/RWA/UFS-NGS-MRC-DPRU442/2012/G1P[8] with cognate gene sequences of RVA/Vaccine/USA/Rotateq-W179-4/1992/G6P1A8.

### Phylogenetic and sequence analyses of VP6, NSP2, and NSP3

Phylogenetically, the VP6, NSP2 and NSP3 gene segments of each Rwandan G1P[8] strain, with the exception of RotaTeq vdG1P[8] strain, segregated or grouped with cognate gene sequences of Wa-like strains belonging to genotype 1 (I1, N1 and T1, respectively) (Fig. 2C,H,I), while those of the RotaTeq vdG1P[8] clustered together with genotype I2, N2 and T6 strains, respectively. Phylogenetic analyses of the VP6, NSP2 and NSP3 nucleotide sequences of the pre- and post-vaccine introduction G1P[8] strains from Rwandan showed a segregation into typical sub-lineages and mixed sub-lineages (consisting of both pre- and post-vaccine introduction strains) (Fig. 2C,H,I). For the VP6 gene, the study strains separated into a post-vaccine sub-lineage and two mixed sub-lineages each consisting of pre- and post-vaccine introduction VP6 genes (Fig. 2C). The nt (aa) similarities amongst the five VP6 genes in the typical post-vaccine introduction strains sub-lineage ranged from 99.3–100% (99.7–100%). The nine I1 genes that grouped in the first mixed pre- and post-vaccine introduction strains sub-lineage revealed gene identities of 98.9–100% (99.5–100%), while the nt (aa) identities of the 21 strains that belonged to the second mixed pre- and post-vaccine introduction strains sub-lineage was in the ranged of 95.1–100% (93.7–100%). The NSP2 genes segregated into three sub-lineages, a pre-vaccine sub-lineage, a mixed pre- and post-vaccine sub-lineage and a post-vaccine sub-lineage (Fig. 2H). Two orphan strains, RVA/Human-wt/RWA/UFS-NGS-MRC-DPRU1575/2011/G1P[8] and RVA/Human-wt/RWA/UFS-NGS-MRC-DPRU669/2013/G1P[8] were also observed (Fig. 2H). The seven genotype N1 strains that clustered in the pre-vaccine introduction strains sub-lineage exhibited nt (aa) similarities of 99.7–100% (100%) amongst each other, while the mixed pre- and post-vaccine introduction strains sub-lineage demonstrated gene identities that ranged from 98.7–100% (98.7–100%). The nt (aa) similarities of the five post-vaccine introduction strains detected in the 2013 RVA season and grouped in a single sub-lineage were 99.7–100% (99.7–100%). For the NSP3, gene sequences of the study strains segregated into one typical post-vaccine introduction strains sub-lineage and one mixed pre- and post-vaccine introduction RVA strains sub-lineage comprising of 8 pre- and 5 post-vaccine introduction study strains (Fig. 2I). The nt (aa) similarities of the NSP3 genes in the typical post-vaccine introduction strains sub-lineage was 99.5–100% (99.1–100%), while those in the mixed sub-lineage had gene identities ranging from 98.4–100% (97.7–100%). Three orphan strains RVA/Human-wt/RWA/UFS-NGS-MRC-DPRU1621/2011/G1P[8], RVA/Human-wt/RWA/UFS-NGS-MRC-DPRU1626/2011/G1P[8] and RVA/Human-wt/RWA/UFS-NGS-MRC-DPRU669/2011/G1P[8] that did not cluster in either of the typical or mixed sub-lineages were observed (Fig. 2I). The VP6, NSP2 and NSP3 gene sequences of the single RotaTeq vdG1P[8] strains shared nt (aa) homology of 98.0%, ≥ 99.7% and 100% with cognate gene sequence of RotaTeq vaccine strains, respectively.

## Discussion

The introduction of the rotavirus vaccines has resulted in the reduction of the global burden of RVA associated diarrhoea diseases and hospitalization^1^. Hence, continuous RVA surveillance in different settings remains vital to document and characterize post-vaccine licensure era RVA strains, possible vaccine derived strains and intergenogroup reassortant strains as part effort to document impact of rotavirus vaccination. Therefore, in the present study, the whole gene analyses of 35 Rwandan G1P[8] and a single RotaTeq vdG1P[8] strains collected from children less than 5 years of age during pre- and post-vaccination with RotaTeq showed that all 35 of the strains possessed a typical Wa-like genotype constellation, while the single RotaTeq vdG1P[8] strain RVA/Human-wt/RWA/UFS-NGS-MRC-DPRU442/2012/G1P[8], was apparently produced by a reassortment event between vaccine strains, RVA/Vaccine/USA/RotaTeq-WI79-4/1992/G6P1A[8] and RVA/Vaccine/USA/Rotateq-W179-9/1992/G1P7[5]. However, the VP4 and VP6 genes may have originated from other circulating vaccine strains as their homologies with vaccine strain RVA/Vaccine/USA/RotaTeq-WI79-4/1992/G6P1A[8] and RVA/Vaccine/USA/Rotateq-W179-9/1992/G1P7[5] were lower than other genes. Whole gene analyses confirmed that the VP7 and VP4 genes were vaccine derived and the RotaTeq vaccine WC3 bovine genetic backbone was confirmed to be G1-P[8]-I2-R2-C2-M2-A3-N2-T6-E2-H3. Though reassortment events between RotaTeq vaccine strains which generated vaccine derived G1P[8] reassortant have been reported previously in Australia, South Korea, Finland and USA, this is the first reported RotaTeq vdG1P[8] from the African continent^34–37^. Previous studies have shown that reassortment may occur more frequently between the VP7 gene of RotaTeq vaccine strain RVA/Vaccine/USA/Rotateq-W179-9/1992/G1P7[5] and VP4 gene of RotaTeq vaccine strain RVA/Vaccine/USA/RotaTeq-WI79-4/1992/G6P1A[8] than between other genotypes^35,36^. The likelihood that this vdG1P[8] may possess increased virulence and cause AGE exists^36^. The combination of the two human outer capsid proteins could potentially enhance cell binding and entry into enterocytes^36^, hence an increase in virulence of this vdG1P[8] strain.

Although inter-genogroup reassortant G1P[8] strains have been reported globally, generally the G1P[8] strains are usually associated with the Wa-like backbone^5,38–41^. Phylogenetically, the 11 gene segments of the Rwanda pre- and post-vaccine introduction G1P[8] strains, with exception of the RotaTeq vdG1P[8], were highly similar and clustered together with cognate gene segments of Wa-like strains (Figs. 1, 2A–K) and further segregated into either typical sub-lineages consisted of only pre-vaccine or post-vaccine introduction strains or mixtures of pre- and post-vaccine introduction strains. The segregation of pre- and post-vaccine introduction G1P[8] strains into different lineages and/or sub-lineages have been previously described in South Africa^20^. In each gene, we observed segregation of strains in terms of vaccination status and year of detection. With the exception of the NSP1 gene which showed the 2013, 2015 and 2016 post-vaccine strains in 3 different sub-lineages, the post-vaccine introduction G1P[8] strains detected during the 2015–2016 RVA seasons always clustered together within the post-vaccine introduction sublineages. The VP1–VP3, NSP1, NSP4 and NSP5 gene sequences of the pre-vaccine introduction strains always clustered together. Analyses of the VP7, VP4, VP6, NSP2 and NSP3 gene sequences, however, showed mixed sub-lineages consisting of both the pre-vaccine strains detected in the 2011 RVA season and post-vaccine introduction strains detected in the 2013 (VP7, VP4 and NSP3), and 2015–2016 (VP6 and NSP2) seasons. In addition, within the typical pre- and post-vaccine introduction sub-lineages of all 11 gene segments, the sequence similarity was extremely high ≥ 99.9% and between sub-lineages the nt (aa) similarities were moderately high in the range of 96–98% (98.1–99.2%). The high genetic relationship between gene sequences of the Rwandan pre- and post-vaccine introduction G1P[8] strains is an indication that the same strains that were circulating before vaccine introduction in Rwanda, are the same strains or their progeny that are causing RVA-associated diarrheal diseases and hospitalization after vaccination.

Globally, at least seven lineages have been previously described for G1 strains collected from different geographical locations^25,30,42,43^. The emergence of distinct lineages or sub-lineages is attributed to the diverse evolutionary mechanisms such as mutation, recombination and reassortments^7^. Based on the VP7 gene, the 35 Rwandan G1 strains which consisted of both pre- and post-vaccine introduction strains, clustered in lineage I and were comparable to observation reported previously^20, 22,44^. On the other hand, the VP4 plays a role as an antibody-neutralization protein^45,46^. Diversity of the P[8] genotypes has been demonstrated through four described distinct lineages^30,47^. The clustering of the 35 P[8] strains in lineage III in this study is consistent with previous observations^19, 20,22,44^. However, despite the clustering of all VP7 and VP4 genes of the study strains in Lineage I (Fig. 2A) and lineage III (Fig. 2B), respectively, antigenic variation seen at the neutralization epitope sites of the VP7 and VP4 proteins of the study strains have been previously described in Belgium^48^ and Australia^49^. Amino acid substitutions with effect on polarity changes were detected on Rwandan VP7 neutralization epitopes at positions T91A/V, S190D and M217. Amino acid substitutions on positions 94, 96, 147, 148, 190, 208, 211, 213 and 217 are critical and have been reported to alter rotavirus antigenicity^50,51^. Therefore, the detected substitutions may be involved in antigenic drift in Rwandan G1 strains. The resultant alteration in charge and polarity observed at position T91A/V on the VP7 neutralization epitope may play a role in escape of host immunity as it was distinctively observed post vaccine introduction^24,48^. Furthermore, the change in polarity at position S190D and M217 could be due to natural fluctuation of RVA as they were observed during the pre-vaccine era and consistently before and after vaccine introduction, respectively^38^. The presence of amino acid differences in cytotoxic T lymphocyte epitope positions Y41F/S, V42M and A46V in Rwandan G1 strains possibly can result in greater host-immunity escape effects^28^. In the intestine, trypsin like proteases cleave the VP4 spike protein into two structural domains (VP8* and VP5*)^4^. Four surface-exposed antigenic epitopes (8-1 to 8-4) have been described in the VP8* region, while five antigenic epitopes (5-1 to 5-5) in the VP5* region have been documented^48^. The amino acid changes identified at positions N113D, S131R and N135D in this study, resulted in polarity changes and play a role in escape of host-immunity^32,33^.

Overall, full genomic analyses of 35 Rwandan G1P[8] strains revealed the predominance of G1-I and P[8]-III, which is consistent with what has been reported previously in Belgium^48^. The global prevalence of Wa-like human strains is hypothesized to be due to the ease of dissemination of this genetic backbone in the human host^21,52^. Although this study was insightful in reporting the whole gene composition of the circulating G1P[8] strains in Rwanda, it was limited to a few years (just 1 year) pre- and (5 years) post-vaccination samples were included. Another limitation was that, for the sample where RotaTeq vaccine shedding was observed, other plausible alternate aetiologies such as adenovirus and/or norovirus that might have led to the diarrhoea symptoms and possible hospitalization, were not evaluated. Cell culture was not used to confirm the reassortment events as the project was mainly focused on in-silico work.

In conclusion, this is the first study to describe full genomic analyses of G1[P8] RVA strains in Rwanda. The detection of RotaTeq vdG1P[8] strain from RVA positive child hospitalized with AGE symptoms was unexpected. RVA strain surveillance at whole gene level will enhance further assessment of vaccine impact on circulating RVA strains, the frequency of reassortment events under natural conditions and epidemiological fitness of RVA generated by such events.

## Methods

### Sample collection

RVA positive fecal samples (n = 158) were obtained from children less than 5 years old who were hospitalized with AGE in Rwanda as part of the ongoing WHO AFRO RVA surveillance program. The samples were conventionally genotyped into G and P types at the South African Medical Research Council-Diarrhoeal Pathogens Research Unit (SAMRC/DPRC), a WHO Rotavirus Reference Laboratory in South Africa (WHO RRL-SA). The samples were collected during the pre- (2011–2012) and post- (2012–2016) RVA vaccination periods with RotaTeq vaccine in Rwanda. Collectively, all the samples were stored at − 20 °C at SAMRC/DPRU and G1P[8] strains were selected for whole genome sequencing at the University of the Free State-Next Generation Sequencing (UFS-NGS) Unit.

### Double-stranded RNA extraction and purification

The extraction of dsRNA was performed at the MRC-DPRU and involved a method previously described by Nyaga et al. (2018)^53^. Briefly, a 100 mg stool sample was added to 200 µL freshly made phosphate buffered saline (PBS) (Sigma-Aldrich, Germany). A 900 µL volume of TRI-REAGENT-LS (Molecular Research Center, Inc, Cincinnati, OH, USA) was added to the suspended stool sample to homogenize as well as lyse the cells and cell components. A volume of 300 µL chloroform (Sigma-Aldrich, Germany) was then added and centrifugation (13,000 rpm for 20 min at 4 °C) was done in a temperature-controlled microcentrifuge (Eppendorf centrifuge 5427R, Hamburg, Germany). The supernatant containing the total RNA was precipitated by addition of 700 µL isopropanol (Sigma-Aldrich, Germany) and by centrifugation at 16,000×*g* for 30 min at room temperature. The resulting pellet was re-dissolved by addition of 90 µL of ddH20 (Merck KGaA, Germany). A concentration of 8 M LiCl_2_ (Sigma, St. Louis, MO, USA) was used to remove ssRNA through precipitation for 16 h after which further centrifugation was done for 30 min at 16,000×*g*. The extracted dsRNA was purified by utilizing the MinElute gel extraction kit (Qiagen, Hilden, Germany) and the integrity and enrichment of the dsRNA was verified via agarose gel electrophoresis.

### cDNA synthesis

Complementary DNA (cDNA) was generated from the extracted viral RNA utilizing Maxima H Minus Double-Stranded cDNA Synthesis Kit with minor modifications (Thermo Fischer Scientific, Waltham, MA, USA). Briefly, the extracted total RNA was denatured at 95 °C for five minutes and then 1 µL random hexamer primers were added. The hexamer primers were allowed to anneal at 65 °C for 5 min. A volume of 5 µL of First Strand Reaction Mix and 1 µL of First Strand Enzyme Mix was then added. The solution was then incubated at 25 °C, 50 °C and 85 °C for 10, 120 and 5 min, respectively. The tubes were removed from the thermocycler and second-strand synthesis was performed by adding 55 µL nuclease-free water, followed by addition of 20 µL of 5 × Second Strand Reaction Mix and 5 µL of Second Strand Enzyme Mix. Subsequently, the solution was incubated at 16 °C for one hour and the reaction was stopped with 6 µL 0.5 M EDTA. Residual RNA was removed with 10 µL RNase I and the synthesized cDNA was incubated at room temperature for 5 min.

### DNA library preparations and whole genome sequencing

DNA libraries were prepared using the Nextera XT DNA Library Preparation Kit (Illumina, San Diego, CA, USA) following the manufacturer’s instructions. Briefly, DNA library preparation entailed tagmentation of the generated DNA, indexing using unique barcodes and amplification of tagmented DNA and clean-up of the amplified DNA. The library quality and size was assessed using an Agilent 2100 BioAnalyzer (Agilent Technologies, Waldbronn, Germany) according to the manufacturer’s specified protocol. The Illumina custom protocol was utilized to normalize the libraries to 4 nM. All the normalized libraries were then pooled together into a single tube by combining 5 µL of each barcoded library. The pooled libraries were subjected to chemical denaturation using 0.2 N sodium hydroxide. After denaturation, 990 µL of pre-chilled hybridization buffer HT1 (Illumina) was added to the 10 µL of the 4 nM denatured DNA library to dilute to 20 pM. A further dilution of the denatured library was performed to get the desired final concentration of 8 pM. A PhiX control spike-in of 20% was used. Whole genome sequencing was performed for 600 cycles (301 × 2 paired-end) on a MiSeq benchtop sequencer (Illumina, San Diego, California, USA) using Illumina V3 reagent kit at the UFS-NGS Unit, Bloemfontein, South Africa.

### Genome assembly

Illumina sequence reads were analyzed using Geneious software v11 (https://www.geneious.com)^54^ and CLC Genomics Workbench v11 (CLC Bio, Qiagen) which entailed genome assembly and mapping the reads to reference-based sequences to obtain the full-length genomes.

### Identification of genotype constellations

Genotyping was performed by utilizing RotaC, v 2.0^55^, an automated online genotyping tool for group A RV strains. Genome constellations were generated by assigning genotypes to each genome segments.

### GenBank accession numbers

The sequences were deposited into GenBank under the accession number MN632673-MN633067.

### Phylogenetic, sequence analyses and protein modelling

For each gene segment, the ORF were aligned and subjected to sequence comparisons as described previously^56–58^. Briefly, multiple alignments were made using the MUSCLE algorithm implemented in MEGA 6 software (https://www.megasoftware.net/)^59^. Once aligned, the DNA Model Test program implemented in MEGA version 6 was used to identify the optimal evolutionary models that best fit the sequence datasets. Using the Corrected Akaike Information Criterion (AICc), the model GTR-G-I was found to best fit the sequence data for each gene segment. With this model, maximum likelihood trees were constructed using MEGA 6 with 1000 bootstrap replicates to estimate branch support. The nucleotide and deduced amino acid sequence identities among strains were calculated for each gene using distance matrices prepared using the p-distance algorithm in MEGA 6 software^59^. Protein modelling was performed on amino acid sequences for each strain and reference strain using the Swiss-Model protein structure homology-modelling server (https://swissmodel.expasy.org)^60^. The structures were modified using UCSF Chimera^61^.

### Ethics

The protocol used in this study was reviewed and approved by the Health Sciences Research Ethics Committee (HSREC) of the University of the Free State under the following clearance number: UFS-HSD2019/1601/2810.

### Disclaimer

The findings and conclusions in this report are those of the authors and do not represent the official position of the World Health Organization and of the Centers for Disease Control and Prevention. Names of specific vendors, manufacturers, or products are included for public health and informational purposes; inclusion does not imply endorsement of the vendors, manufacturers, or products by the World Health Organization, Centers for Disease Control and Prevention or the US Department of Health and Human Services^38,62–64^.
